# A Comparative Review of Thermocouple and Infrared Radiation Temperature Measurement Methods during the Machining of Metals

**DOI:** 10.3390/s22134693

**Published:** 2022-06-22

**Authors:** Emilios Leonidas, Sabino Ayvar-Soberanis, Hatim Laalej, Stephen Fitzpatrick, Jon R. Willmott

**Affiliations:** 1 Department of Material Science & Engineering, University of Sheffield, Mappin Street, Sheffield S1 3JD, UK; eleonidas1@sheffield.ac.uk; 2 Sensor Systems Group, Department of Electrical & Electronic Engineering, University of Sheffield, Portabello Centre, Pitt Street, Sheffield S1 4ET, UK; 3 Advanced Manufacturing Research Centre (AMRC), Machining Research, Process Modelling & Control Group, Factory of the Future, Wallis Way, Advanced Manufacturing Park, Catcliffe, Rotherham S60 5TZ, South Yorkshire, UK; s.ayvar@amrc.co.uk (S.A.-S.); h.laalej@amrc.co.uk (H.L.); 4 Advanced Forming Research Centre (AFRC), Advanced Forming Research Centre, 85 Inchinnan Drive, Paisley PA4 9LJ, UK; s.fitzpatrick@strath.ac.uk

**Keywords:** metallurgy, temperature, measurement, monitoring, machining, thermocouples, infrared radiation thermometer

## Abstract

During the machining process, substantial thermal loads are generated due to tribological factors and plastic deformation. The increase in temperature during the cutting process can lead to accelerated tool wear, reducing the tool’s lifespan; the degradation of machining accuracy in the form of dimensional inaccuracies; and thermally induced defects affecting the metallurgical properties of the machined component. These effects can lead to a significant increase in operational costs and waste which deviate from the sustainability goals of Industry 4.0. Temperature is an important machining response; however, it is one of the most difficult factors to monitor, especially in high-speed machining applications such as drilling and milling, because of the high rotational speeds of the cutting tool and the aggressive machining environments. In this article, thermocouple and infrared radiation temperature measurement methods used by researchers to monitor temperature during turning, drilling and milling operations are reviewed. The major merits and limitations of each temperature measurement methodology are discussed and evaluated. Thermocouples offer a relatively inexpensive solution; however, they are prone to calibration drifts and their response times are insufficient to capture rapid temperature changes in high-speed operations. Fibre optic infrared thermometers have very fast response times; however, they can be relatively expensive and require a more robust implementation. It was found that no one temperature measurement methodology is ideal for all machining operations. The most suitable temperature measurement method can be selected by individual researchers based upon their experimental requirements using critical criteria, which include the expected temperature range, the sensor sensitivity to noise, responsiveness and cost.

## 1. Introduction

Metal machining creates substantial thermal and mechanical loads that can impact the mechanical behaviours of both tools and workpieces [1,2,3,4,5,6,7]. However, the machining industry focuses more on the mechanical loads of the operations. For example, one of the most common mechanical parameters is the cutting force [8,9]. Measuring the cutting force, tool geometry and analysing chip formation allows for the estimation of operating temperature and tool wear, amongst other factors. The accurate and reliable determination of the temperature, and temperature distribution, in the areas around the cutting tool–workpiece interface using cutting forces can be challenging [10].

Astakhov et al. [11] estimate that 15% of the value of all mechanical components manufactured worldwide is derived from machining operations. With the manufacturing industry moving away from the use of lubricants and coolants due to their effects on the environment, reliable temperature measurements during machining operations are becoming increasingly important [12]. In addition, machining performance in terms of cutting forces, surface roughness and tool life, amongst others, is directly linked with temperature [13]. Therefore, in-situ temperature sensing and monitoring aids in realising Industry 4.0 goals.

A review article by Childs et al. [14] reviewed the general temperature measurement methodologies available in terms of their accuracy, thermal disturbance and calibration. The temperature measurement methodologies described are not specific to one application, but some of the methodologies are applicable for machining operations. Their work also provides an insight into the background theory for each methodology. Davies et al. [15] reviewed the methodologies applicable for material removal processes, giving a historical overview of the advancements in temperature measurement for material removal processes. The authors provided a brief overview on the background theory for each methodology and proceeded to discuss major work performed in various material removal processes, highlighting the capabilities and challenges faced in each process. The study by Pimenov et al. [16] reviewed literature for tool-condition monitoring systems, including systems for temperature measurements, used with artificial-intelligence algorithms. The study by Pashnyov et al. [17] reviewed mathematical models for temperature distribution during grinding operations of metal-composite structures followed by analysis of metal composite systems on the basis of these models to identify the influence of lamination on the nature of temperature distribution. The work by Kuntoğlu et al. [9] reviewed methodologies for indirect tool-condition monitoring systems for turning operations. A later study by Kuntoğlu et al. [18] reviewed sensors and signal monitoring systems in machining processes, which briefly included temperature measurement systems. This is a valuable resource for signal conditioning and processing. Korkmaz et al. [13] reviewed literature on online detection methods and signal processing systems used in machining, including some literature for cutting temperature online monitoring systems. Zhao et al. [19] reviewed the temperature measurement methodologies employed by researchers investigating the effect of tool coatings on cutting temperature. Their work was focused on predictive modeling with coated cutting tools and only discussed temperature measurement methodologies used by researchers when experimentally validating their models. Therefore, the methodologies discussed in their work were limited and not representative of the temperature measurement methodologies found in the literature.

The focus of this article is to review the latest advancements in temperature measurement methodologies for metal turning, drilling and milling operations, with emphasis given to the methodologies’ applicability in industrial or research environments. Our research methodology, therefore, focused on the following search terms: “temperature”, “measurement”, “machining”, “turning”, “milling”, “drilling”, “metal cutting”, “monitoring”, “thermocouple”, “pyrometry”, “infrared radiation thermometer” and “thermometry”. These terms were used to search three online scientific citation indexing services (Web of Science, Scopus, and Google Scholar) in order to obtain the articles that make up this review. These databases were used to elucidate the key researchers that have published in the aforementioned categories of temperature measurements for metal machining. This allowed us to see the current leading edge in the field, by seeing where the research leaders were most active. The second strand to our method was to build an understanding of the influence of temperature on the workpiece and the tool, as well as how heat is generated and partitioned. Heat generation and partitioning enables researchers to understand heat distribution within the material in order to identify areas of interest to measure their temperature. These are described below and we have conjoined our understanding of heat generation and distribution with our review of the state-of-the-art methods. As our focus is on thermocouple- and radiative-temperature measurement methodologies in the turning, drilling and milling of metals, abrasive machining and other temperature measurement methodologies have largely been excluded from this work. Whilst other temperature measurement methodologies, such as thermal paints and PVD coating, have been found in the literature, they do not fit the scope of this article because they are not very accurate and are prone to errors. It is also worthwhile mentioning that the temperature measurement methodologies can also be applied for the machining of non-metallic materials. In this review, we have provided a comprehensive repository for information on the different thermocouple and radiative temperature measurement methodologies applied to metal machining operations; by whom the leading research is being conducted; the nature of the research; and our interpretation of how the research fits within the overall research field.

### 1.1. Temperature during Machining

The importance of the effect of cutting temperature has been recognised since 1907, when F.W. Taylor [20] was the first to demonstrate the relationship between tool life and cutting speed, and thus the cutting temperature. Ever since Taylor’s work, there has been a stimulus to identify methods of measuring the cutting temperatures during machining. The manufacturing industry, especially the machining industry, has shown great interest in knowing and understanding how heat is generated and distributed during cutting processes [21,22]. Abukshim et al. [23] stated that the machining of metals is still not completely understood due to the highly non-linear nature of the process and the complex coupling between deformation and temperature fields.

During the machining operation of metals, there is a significant increase in temperature at the workpiece–tool interface. Depending on the loading parameters and the metal to be machined (such as titanium, which has a low thermal conductivity [24,25]), the cutting temperatures at the tool–workpiece interface can reach an excess of 1100 °C [26], with the most common temperatures in general machining in the range of 500 °C to 1000 °C [22,27]. This interfacial temperature increase comes from friction and the plastic deformation energies being transformed to heat [28,29,30]. Some of these influences include [31,32,33,34,35,36,37]:

- Acceleration of tool wear and subsequently reducing lifespan, therefore increasing operational costs.
- Thermal deformation of the work piece, cutting tool and machine tool leading to degradation in machining accuracy. This is mainly observed in the form of dimensional inaccuracies due to thermal distortion as well as expansion and contraction on the workpiece during and after machining.
- Subsurface layers of the workpiece are destabilised through phase transformation, residual stresses as well as other thermally induced defects that affect the metallurgical properties of the machined component. This can lead to the introduction of residual tensile stresses and micro cracks at surface and subsurface levels, as well as cause surface damage via oxidation and corrosion.

With the industry moving towards dry and cryogenic machining for a reduction in its carbon footprint [38], as well as for environmental reasons linked with the use of lubricant coolants [39,40], temperature monitoring at the workpiece–tool interfaces is becoming increasingly paramount [41]. A critical analysis of the ecological aspects of various machining conditions was performed by Krolczyk et al. [42]. Temperature is an important parameter in machining, if not the most important [31], and it can have several influences on the machining outcome. The machining operation can be more effectively controlled by obtaining accurate and reliable temperature readings, resulting in a more efficient process. The aforementioned reasons signify the importance of the role of temperature on the outcome of machining processes. Therefore, they indicate the need for further development in the field of temperature measurement to further our understanding of metal machining.

### 1.2. Heat Generation during Machining

It is necessary to have an in-depth understanding of the factors contributing to heat generation and distribution during machining operations for metals. This is an essential element in tool development and process optimisation. It is known that, during metal machining, significant quantities of heat are generated due to tribological factors and plastic-deformation energy transformations [28,29,30]. Trent and Wright [10] suggested that 99% of the work performed during machining is converted to heat. Therefore, it is assumed that nearly all of the energy due to tribological factors and plastic deformation, during chip or swarf formation, is converted to heat, which is observed as a temperature rise in the cutting zone [3].

**Figure 1:**
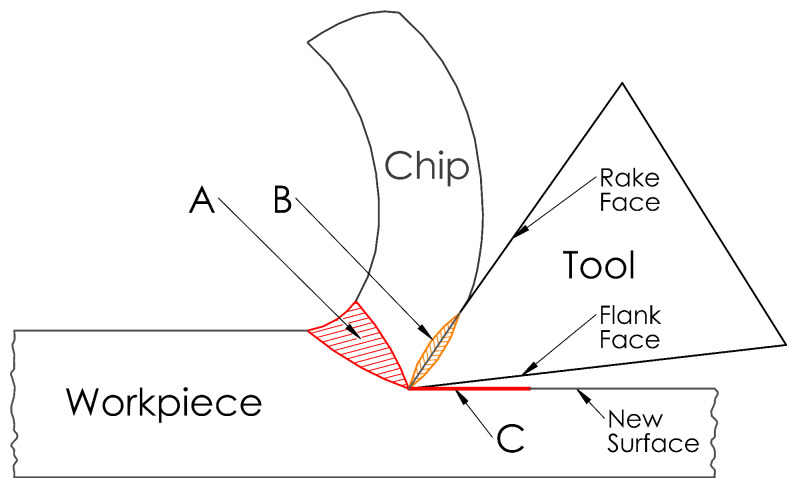
Sources of heat generation during metal cutting.

There are three main regions where heat is generated in the cutting zone, as schematically shown in Figure 1.

- Region A is the primary shear, or deformation, zone. In this region, the workpiece material is subject to shearing and plastic deformation to form a chip. The majority of the energy released during plastic deformation is converted into heat. The heat generated in this region is transferred to the chip and the workpiece [10,43].
- Region B is the secondary deformation zone which lies on the tool–chip interface. Heat is generated due to the deformation of the chip material and tribological factors on the tool rake face as the chip material overcomes both the adhesive and the sliding friction as it separates from the tool–chip interface [43,44]. This interface is where the maximum heat is encountered [22,45].
- Region C is the tertiary deformation zone, or tool–workpiece interface, where the tool flank moves along the newly formed workpiece surface with heat generation influenced by tribological factors. The geometry of the cutting tool is the main factor affecting how much heat will be generated in this region. To reduce friction in this region, the cutting tool provides a clearance angle between the workpiece and the flank surface, typically ranging between 3° and 15° [46,47,48]. As heat generation due to friction in this region is dependent on the tool geometry, more heat is generated as the tool wears [49], which can significantly impact the surface quality of the workpiece [45].

Heat generation during metal machining is dependent on combination of the physical and chemical properties of the workpiece and cutting-tool materials (including cutting-tool coating), machining parameters and cutting-tool geometry. In general, the amount of heat generated, and hence the temperature, in the primary and secondary deformation zones is dependent on the material properties and the machining parameters [10,50]. In contrast, the amount of heat generated in the tertiary deformation zone is mainly dependent on the geometry of the tool flank [43,49].

## 2. Temperature Measurement Methods

Many research studies have been conducted to find a reliable way to monitor the cutting temperature during machining; however, none of the research solutions have been implemented by the industry. The reason for this is mainly due to difficulties associated with appropriate sensor selection for any given machining operation. The main criteria to be considered for a temperature measurement technique are [14,51,52]:

1. The expected temperature range to be monitored;
2. The sensor robustness to withstand the machining environment conditions;
3. The sensor response time to temperature changes;
4. The sensor’s sensitivity to electrical noise;
5. Temperature field disturbances of the sensor;
6. Cost.

**Figure 2:**
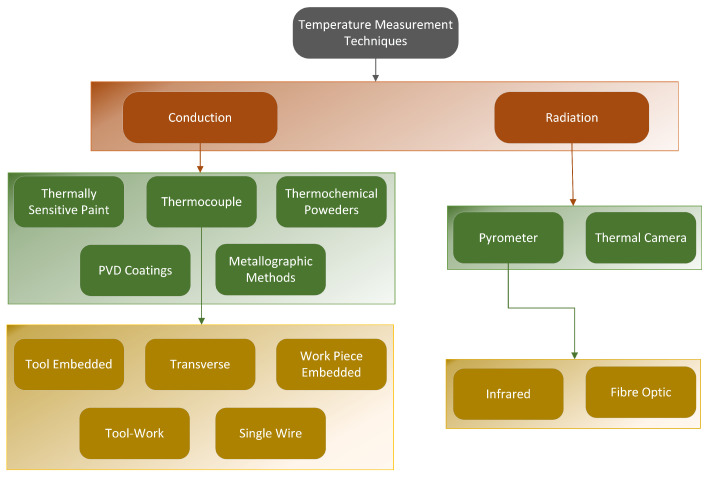
Temperature measurement techniques based on Byrne [31].

The temperature monitoring techniques found in the literature fall into two main categories: contact (or conductive) and non-conduct (or radiative) techniques, as summarised in Figure 2 [31,53]. Detailed summaries of background theory of these methods and their uncertainties were provided by Childs [51] and Nicholas and White [54], with Heeley et al. [55] providing an uncertainty analysis for their custom fibre-optic infrared thermometer.

### 2.1. Contact Temperature Measurement Methods

Contact, or conductive, techniques measure the temperature gradient by monitoring the heat transfer between two points that are in direct contact with the material to be measured [56]. The thermal paint approach is one of the simplest and more economic temperature measurement techniques available [4]. It requires the material to be coated with the paint, which changes colour depending on the temperature. PVD (physical vapour deposition) coating techniques help determine the internal temperature of the material by melting once the material temperature exceeds a threshold. Thermochemical powders work in a similar way to PVD coating, relying on visual changes resulting from the melting of the powders. One of the main limitations of the techniques mentioned so far is that they are not suitable for more aggressive machining operations, where cutting coolants can remove the coatings from both tool and workpiece.

Metallographic methods utilise a more theoretical approach that correlates metallurgical changes in the workpiece, or tool, to the temperature to which it was exposed, based on existing reference models of the micro structure–temperature relation [31]. However, this technique is limited to certain materials and is unsuitable for common cutting-tool materials, as well as being predisposed to inaccuracies due to difficulties related to acquiring accurate theoretical models. The techniques mentioned so far have a several limitations, rendering them unsuitable for the temperature monitoring during machining applications.

The use of thermocouple sensors is the most common conductive temperature measurement technique [57,58]. The principal factors for thermocouple use is that they are durable, can be relatively inexpensive, and are able to operate over a wide range of temperatures [14,15]. There are several ways to implement thermocouples to obtain temperature measurements during machining, as shown in Figure 2. They could be embedded in the workpiece or tool material for single point measurements, or positioned in various points to obtain a temperature distribution [31]. A limitation common among most thermocouple types is their relatively slow response time, making it difficult to measure quick changes in high levels of temperature, especially in high-speed milling applications. Additional limitations include that thermocouples are known to drift over time due to homogeneity changes at the thermocouple junction at elevated temperatures, as well as hysteresis effects during thermal cyclic conditions, such as the ones observed during milling [59,60]. In a study by Pavlasek et al. [59], it was found that thermocouples drifted at a rate of around 0.22 °C per hour. These limitations would result in a reduction in accuracy with each measurement.

#### 2.1.1. Tool–Workpiece Thermocouples

**Figure 3:**
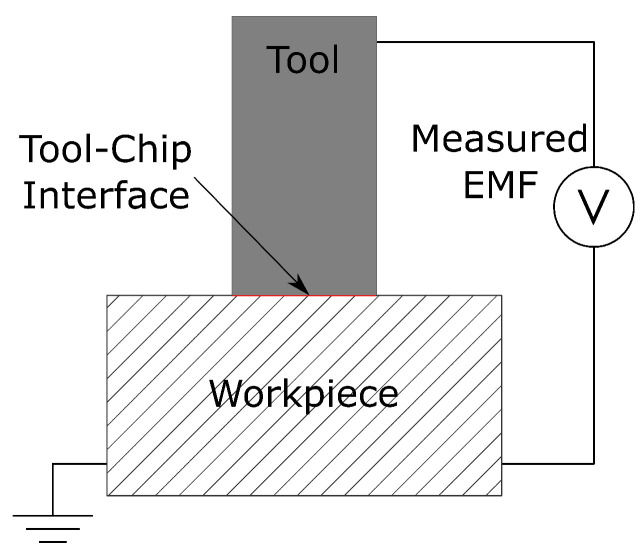
Simplified tool–workpiece thermocouple circuit, based on Stephenson [61].

The tool–workpiece thermocouple method can be used when both the cutting tool and workpiece are electrically conductive and made from dissimilar materials, turning the system into a thermocouple, as shown in Figure 3 [61]. This technique is based on the thermocouple principle, where two dissimilar materials give rise to an electromotive force (EMF) due to a temperature difference between hot and cold junctions [3,62]. The EMF can be measured and correlated with the temperature of the tool–chip interface.

Kitagawa et al. [63] used the tool–workpiece thermocouple technique to investigate the differences in tool life between the dry end milling and turning operations of a titanium alloy under various cutting conditions using a K10 tool. A greater rate of tool-life improvement with increasing cutting speed was observed in end milling than turning. An increase in the mean rake temperature with increasing cutting speeds was also observed.

Grzesik [64] used this technique to investigate the influence of tool coating on cutting temperature during the turning of medium carbon steel and austenitic stainless steel. To obtain reference temperature measurements, a type-K thermocouple was embedded into the workpiece, at a distance of approximately 0.1 mm away from the workpiece surface. To calibrate the thermocouples, a tungsten inert gas (TIG) welding machine was used to heat up the workpiece surface within a small area adjacent to the cutting interface with the measured EMF relating to a temperature. The area heated up by the TIG was used as the hot junction, whereas an ice bath was used as a cold junction. This process was repeated for both workpiece materials with differently coated cutting tools. The main limitation of this approach was that the ice bath used as a cold junction is not ideal for an industrial setting. Furthermore, for any change in the experimental configuration, more calibrations are required.

Recognising this limitation, Abhang and Hameedullah [62] improved on this technique by setting the tool–workpiece interface as a hot junction and remote sections of the tool and workpiece, which were kept at a constant reference temperature, were used as the cold junction. Doing so, a mercury bath was used at the cold junction, which has health risks associated with it.

To avoid the use of liquids, and their inherent limitations, as cold junctions, Santos Jr et al. [65] considered seven different junctions between various components of their experimental setup as cold junctions. The cold junctions were assumed to be at room temperature and the hot junction was the interface between a K15 cemented carbide tool and 1350-O and 7075-T6 aluminium alloys as workpiece materials. The calibration process took place in a furnace alongside a calibrated K-type thermocouple as a reference. This experimental setup was successful at monitoring the surface-temperature response of the two aluminium alloys with respect to cutting speed, depth of cut and feed rate.

A simpler approach was utilised by Ghodam [66,67], where they insulated and directly connected the workpiece and tool junctions to a multi-meter to measure the induced EMF corresponding to the average temperature of the tool tip. The tool–work thermocouple was calibrated using a heating rod and a K-type thermocouple as a reference. This study aimed to evaluate the machining performance of coated and uncoated cutting tools whilst turning an EN8 steel alloy.

Mia and Dhar [68] used a tool–workpiece-thermocouple setup to obtain temperature measurements to validate their predictive neural network model for turning operations of AISI 1060 steel using a tungsten carbide tool. The tool–workpiece thermocouple was calibrated by heating the tool–workpiece junction in a graphite heating block and validating the temperature by using another standard thermocouple. The same thermocouple setup was used by Mia et al. [69] to monitor the cutting temperature during the turning of AISI 1060 steel to investigate the sustainability of various cooling and lubricating conditions. It was found that the MQL system was the most sustainable out of the conditions tested. Mia et al. [70] used the same tool–workpiece approach to measure the cutting temperature during a cryogenic-assisted turning of Ti-6Al-4V. This study aimed to asses the role of loading parameters on temperature to allow for parameter optimisation and to assess the life cycle of cryogenic-assisted turning. It was found that the cutting temperature was minimised with higher cutting speeds and lower feed rates. Furthermore, it was found that the cryogenic turning of Ti-6Al-4V was a more sustainable alternative to dry machining. Future work from these researchers aims to perform life-cycle assessments for more cooling–lubricating machining conditions for more workpiece materials.

**Section Findings:** The tool–work thermocouple technique is relatively simple to implement in laboratory settings; however, it is not practical to be implemented in industrial production environments. Early implementations of this technique used hazardous liquids at their cold junctions, though more recent implementations have demonstrated that other areas of the setup could be used as cold junctions. A combination of the limitations of the tool–workpiece thermocouple technique from the comprehensive study by Stephenson [61] and those identified in the literature are summarised:

- Limited to electrically conductive tool and workpiece materials, which must also be electrically isolated from the machine tool so as to achieve an accurate signal.
- Electrically isolating the tool could cause the machine tool to be dynamically unstable, making chatter generation more probable during heavy cuts at higher speeds.
- Electrically conducting lubricants and cooling fluids cannot be used with this technique.
- Limited to non-indexable tools, as indexable tools could result in secondary EMF signals being generated between the cutting insert and the tool holder, resulting in measurement errors.
- Calibration would be required for all the different combinations of tool and workpiece materials, which can be inconvenient for practical applications.
- The temperature recorded is an average of the entire contact area.
- The thermocouple circuit is calibrated under static conditions.
- Oxide layers tend to form on carbide tools during machining, which affect the temperature readings as these oxide layers would not be considered during calibration.

#### 2.1.2. Embedded Thermocouples

**Figure 4:**
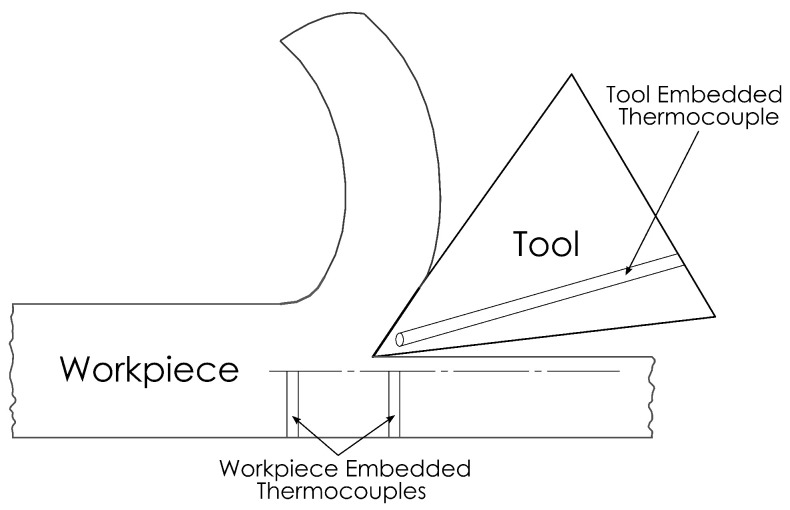
An illustration of tool- and workpiece-embedded thermocouple techniques.

The embedded thermocouple method is perhaps the most widely used approach by researchers for temperature measurements. It is also used to obtain reference values alongside other temperature measurement methods [64,65]. This thermocouple approach has two variations, the tool-embedded and the workpiece-embedded variations; the thermocouple is inserted into a drilled hole in the tool, for the tool-embedded variation, or the workpiece, for the workpiece-embedded variation, at a precise distance from the cutting edge, as illustrated in Figure 4. The hole is typically filled with thermally conductive material, such as ceramic cements, preventing heat losses inside the hole by displacing the air.

Zeilmann and Weingaertner [71] used the workpiece-embedded thermocouple approach to measure the temperature during a minimum quantity lubrication (MQL) drilling of Ti-6Al-4V with cutting speeds between 10 to 40 m/min. K-type thermocouples were embedded into holes in the workpiece at a distance of 0.2 mm from the edge of the hole that was to be drilled into the workpiece. Maximum temperatures of around 200 °C, 280 °C and 320 °C at cutting speeds of 15, 30 and 40 m/min were reported. These findings were compared with those of external MQL drilling and it was found that the temperatures obtained with internal lubrication were 50% lower than those obtained through external lubrication.

Le Coz et al. [72] used the tool-embedded thermocouple approach to measure the cutting tool temperature during internal MQL drilling operations of Ti-6Al-4V. A K-type thermocouple was embedded inside 20 mm deep holes close to the corner of 10 mm diameter drills with internal lubricant feeds. The thermocouple signals were amplified by a transmitter integrated inside the tool holder. A radio frequency antenna was positioned close to the tool holder to allow for high-speed data acquisition. The results obtained by Le Coz et al. [72] were compared to those obtained by Zeilmann and Weingaertner [71] and were found to be much higher, ranging from 590 °C to 640 °C. The explanation for this was that the temperatures obtained through the workpiece-embedded approach were at a distance of 0.2 mm from the wall of the drilled hole, where the tool-embedded approach was able to record the temperature at the tool–workpiece interface.

Bagci and Ozcelik [73,74] employed the tool-embedded thermocouple approach in a novel way by utilising the existing coolant hole on TiAlN-coated carbide drills to measure temperature during the drilling of AISI1040 steel and Al 7075-T651. A PFA Teflon-coated type-K thermocouple was attached in an unspecified manner inside the coolant hole of the drill. The experimental setup kept the drill stationary and the workpiece rotating to simplify data acquisition. In their first study, the effect of drill depth, spindle speed and feed rate on the drill-bit temperature was investigated during step and continuous drilling. In the second study, the same methodology was used to validate their finite-element model.

Ay and Yang [75] used the tool-embedded thermocouple variation to analyse the temperature variations of the tool and workpiece during orthogonal turning of various materials. A triangular carbide tool embedded with nine type-K thermocouples, three at each surface of the cutting edge, was used to machine copper, cast iron, aluminium 6061 and AISI 1045 steel as workpiece materials. They observed oscillations in temperature near the cutting edge that were more rigorous for ductile materials than in hard-to-machine materials.

Hamzawy et al. [76] used the workpiece-embedded thermocouple approach to monitor the workpiece temperature during the friction drilling of 4 mm thick Al-6082 and Al-7075-T6 sheets under various loading parameters. Four type-K thermocouples were positioned at distances of 10, 12, 20 and 24 mm from the centre of the drilled hole to observe the heat distribution within the workpiece materials. The findings of this study included that the temperature increased with increasing rotational speed and tool cone angle, whereas a decrease in temperature was observed with an increased feed rate.

The workpiece-embedded thermocouple approach was also employed by Uçak and Çiçek [77] to investigate the effect of cutting conditions on temperature and hole quality during the drilling of Inconel 718 using solid carbide drills. Five thermocouples were positioned 0.1 mm away from the wall of the drilled hole, equally spaced at a depths of 1.5 mm to 13.5 mm. The experiments were repeated under dry, cryogenic-cooling and wet conditions. Cryogenic drilling was shown to have the greatest impact on reducing the temperature, however, it was shown to greatly reduce tool life. Dry drilling was found to lead to the highest cutting temperatures as well as the greatest tool wear rate. Wet conditions resulted in better surface roughness and better tool life and more stable machining was observed compared to both cryogenic and dry conditions.

O’Sullivan and Cotterell [30,34] utilised the workpiece-embedded thermocouple technique to attach two type-K thermocouples inside an Aluminium 6082-T6 tube. The thermocouples were positioned 4 mm away from the new surface created in a continuous turning operation, and were placed 20 mm and 80 mm from the tube end. A mercury rotating slip ring was used to enable the thermocouple connection, as the thermocouples were rotating with the workpiece. An increase in tool wear was observed with increasing temperatures at the new surface. This approach is hazardous and unsuitable for use in industrial applications because of the health factors associated with mercury.

Ren et al. [78] used a slightly different approach, where a thin type-K thermocouple was inserted in a groove between the shim and the tool. Their experiments aimed to evaluate the average cutting temperature developed at the interface between the shim and the PCBN cutting-tool insert during turning operations of titanium alloy and chromium hard facings. The obtained thermocouple data were used to inform a finite-element (FE) model to reverse calculate the temperature at the tool–chip interface.

Il et al. [79] utilised the workpiece-embedded thermocouple approach to measure the subsurface temperature on an Aluminium 2024-T3 alloy during milling operations with a four flute DLC-coated end-mill of 19.05 mm in diameter. The aim of this study was to investigate the influence of feed per tooth, spindle speed, depth of cut and radial width of cut on the subsurface temperature of the workpiece. The type-K thermocouples were placed inside a blind hole which was filled with thermal cement to secure the thermocouple position and to ensure that there was heat transfer from the workpiece to the thermocouple. From this study, it was found that the optimal milling conditions were achieved for a low depth of cut, high cutting speed and a feed per tooth of at least 0.127 mm.

A similar implementation was used by Akhil et al. [4] and Gosai et al. [80]. In both studies, the thermocouple measurements were used for process optimisation. Krishna and Reddy [81] employed the tool-embedded thermocouple variation to measure the temperature at the tool chip interface using a K-20 carbide tool whilst performing orthogonal turning of aluminium 6061-based metal-matrix composites. The thermocouple data were correlated to an FE model with an observed variation of 1.25%, and concluded that the maximum temperature observed occurred at the tool-chip interface. A similar methodology was employed by Kus et al. [22], where tool-embedded thermocouple measurements were used to inform an FE model.

A more advanced implementation of the tool-embedded thermocouple was used by Kryzhanivskyy et al. [82,83]. Their experiments included eight thermocouples embedded at different locations within the cutting tool to inform their FE model with heat flux as a parameter.

**Section Findings:** The embedded-thermocouple technique is widely used for research in laboratory settings, due to its relatively low cost and simple implementation. However, more research on improving the implementation of this thermocouple technique may be required before it is practical to be implemented in industrial production environments. The workpiece-embedded thermocouple technique can be used where knowledge about heat distribution within the workpiece material is required. This would require several precise holes to be drilled into the material, to accommodate the thermocouples, which can lead to a significant increase in system costs. This technique is useful for obtaining measurements to inform FE models for improved temperature predictions.

The tool-embedded thermocouple technique only requires one hole to be drilled, into the tool. This is more desirable, as there is no additional machining required on the workpiece and it, thus, could be used for industrial environments. Embedded thermocouples provide more accurate measurements compared to other thermocouple techniques, especially in continuous turning operations. The implementation of embedded thermocouples in milling operations is not as straight forward. The limitations of the embedded-thermocouple technique include the following:

- The drilling of a large number of holes may lead to inaccurate results as a consequence of uneven temperature distribution.
- Surface temperature cannot be directly measured with the embedded-thermocouple technique. It can be extrapolated from the temperature measured by the thermocouple deeper within the metal.
- There could be a great increase in cost associated with the difficulty of drilling holes in certain hard-to-machine materials, especially when employing the workpiece-embedded thermocouple technique.
- The thermocouple response time might not be sufficient to measure sudden temperature changes in high-speed milling operations due to the short contact time of the tool with the workpiece.

#### 2.1.3. Single Wire Thermocouples

**Figure 5:**
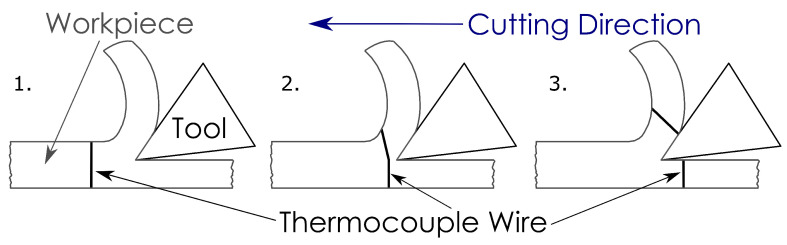
Illustration of single-wire thermocouple machining sequence.

The single-wire thermocouple technique, also referred to as a semi-artificial thermocouple, is a variation of the workpiece-embedded thermocouple technique. This technique requires an insulated thermocouple wire, typically a constantan wire, to be fitted inside the workpiece with the temperature measured as the wire is cut with the workpiece material, as illustrated in Figure 5. The workpiece is required to be sliced in two across the line of cutting for the wire to be fitted inbetween the two halves. This technique was developed by Black et al. [84] to measure the temperature distributions during both wet and dry grinding operations, and has been demonstrated for use in other machining operations.

This technique was used by Dewes et al. [85] to measure the tool–workpiece temperature during dry milling using a ball nose tool at an angle of 0° and 60° relative to the workpiece. Higher temperatures were observed when machining at 60° than at 0°.

Sun et al. [86] used this technique to measure the temperature of a titanium alloy (Ti-6Al-4V) as it was machined. It was assumed that the highest temperature recorded corresponded to the point when the cutting tool first made contact with the workpiece. Therefore, correlating the recorded temperature to the specific instances during the milling process to obtain the temperature at the tool–chip interface as well as the workpiece temperature.

In another study, Baohai et al. [87] obtained temperature measurements to verify their temperature FE model during milling operations of Inconel 718. Both the experimentally obtained measurements and the data obtained from the FE model followed the same trends. However, the experimental measurements appeared to be lower than the equivalent data from the FE model because of experimental constraints not accounted for in the FE model.

**Section Findings:** This technique is suitable for experimental work because it can aid in validating FE models for temperature predictions. However, as a consequence of the workpiece requiring to be sliced, this technique is not suitable for industrial applications. The single-wire thermocouple technique is subject to the following limitations:

- The thermocouple wire needs to be replaced after each cutting operation, which can lead to a significant increase in cost.
- There are additional costs in the manufacturing process by having to calibrate the thermocouple system each time the wire is replaced, as it is not a standard thermocouple.
- Additional machining is required on the workpiece to fit the thermocouple wire, which has a detrimental impact on the structural integrity of the workpiece, greatly increasing machining-associated costs.
- The maximum temperature at the tool–chip interface is not always recorded due to experimental errors introduced by variations in detecting the tool position along the cutting edge. To overcome this limitation, additional replications are required, which lead to an increase in the overall cost.
- Not suitable for turning operations as a result of additional complexities introduced attempting to fit the sensor electronics on a workpiece in constant rotation.
- Drilling a hole into the cutting tool could affect its structural integrity.

### 2.2. Radiative Temperature Measurement Methods

Non-contact, or radiative, measurement techniques interpret and measure the thermal energy, in the form of infrared (IR) radiation, emitted by an object of interest to determine its thermodynamic temperature based on the wavelength of the emitted radiation [15,88]. This often allows for non-intrusive temperature measurements to be taken from a distance [56]. Thermal, or infrared, cameras are widely used in milling applications [30,34], as they can record the surface temperature gradient over a large area of the workpiece and tool in the form of live thermal images.

Infrared thermometers, which are often referred to in the literature as pyrometers, operate on the same principle as thermal cameras but only record a single temperature measurement over their field of view. Infrared thermometers (IRTs) have two basic configurations. The first configuration consists of focusing optics and a detector, with the IRT recording the average temperature of the surface within its field of view. This configuration of IRTs and thermal cameras requires a direct line of sight to the workpiece or tool surface that is measured. The second configuration includes an optical fibre to transmit the infrared radiation emitted from a source to the detector. Instruments with this configuration are usually referred to as fibre optic infrared thermometers (FO IRTs). FO IRTs enable the processing electronics to be positioned away from aggressive environments, such as the one encountered during machining. FO IRTs can be embedded, such as the case with tool- and workpiece-embedded thermocouples, as well as used in a non-intrusive manner, operating in a similar way to an IRT.

#### 2.2.1. Infrared Cameras

**Figure 6:**
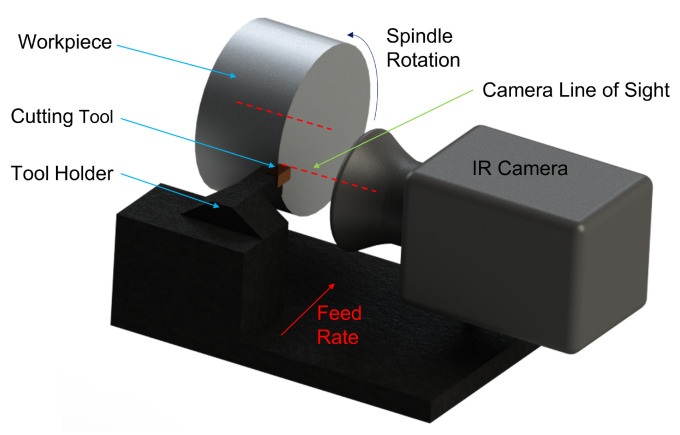
An illustration of a simplified typical infrared-camera setup.

The use of infrared cameras for determining the distribution in the temperature during machining operations was developed by Boothroyd [89,90] in 1961. Boothroyd used a photographic plate that required an exposure time of 15 s to obtain an image. However, thermal-imaging technology has gone through a large transformation through the advancement of digital-camera sensors. Thermal cameras are now capable of providing live thermal images of the process at very high frame rates. For an IR camera to obtain the area measurement, it needs to have a direct line of sight to the area of interest, as illustrated in Figure 6.

Danish et al. [91] used a FLIR T640 thermal camera, with a set emissivity value of 0.18, to investigate the temperature at the machined surface during dry and cryogenic turning operations of a AZ31 magnesium alloy. During the calibration process of the camera, a sample was heated on a hot plate from 20 °C to 200 °C with the measurements validated with the use of a thermocouple attached to the workpiece at the area observed by the camera. It is important to note that, in this study, one emissivity value was set for both dry and cryogenic conditions, which might not reflect the real values of emissivity for both conditions. The findings of these experiments were used to validate their developed finite element model with a maximum percentage error of 7%.

Satur et al. [92] used a 240 × 180 pixel infrared camera by Testo company, to investigate the effects of MQL and dry environments during the end milling of AISI 1040 steel on cutting temperature, tool wear and power consumption. The thermal camera was positioned approximately 50 cm from the cutting zone and considered only the peak temperature values pixel by pixel from the interface. It was observed that the cutting temperature, tool wear and power consumption yielded better results for MQL environments compared to dry environments.

In the study by Gupta et al. [93] a Fluke thermal camera was used to measure the temperature at the primary shear zone with the purpose of experimentally validating their predictive model for the cryogenic-assisted turning of AA2024-T351. The main challenge faced during the experimental validations was that the camera was observing the back of the newly formed chip, whereas their model was designed to calculate the temperature at the tool–chip interface. To overcome this challenge, initial experimental data were taken five times to calibrate their results against modeling data. This enabled the extrapolation of the interfacial temperature from measuring the back surface of the chip.

Dewes et al. [85] used an IR camera sensitive to the spectral range of 8–12 µm alongside the single-wire thermocouple technique to measure the tool–chip temperature in dry milling. Lower temperatures were recorded with the IR camera than the single wire thermocouple, which were explained due to variability in emissivity as well as the fact that the camera observed the back side of the formed chip. An IR camera was employed by O’Sullivan et al. [30,34] to determine the temperature of the machined surface as a means to verify their tool-embedded thermocouple results. Decreased surface temperature was observed for higher cutting speeds, and increased tool-flank wear led to higher surface temperatures. Young [94] measured the temperature of the back of the chip, as well as the interfacial temperature, to investigate the effect of tool wear on temperature, during the orthogonal cutting of an AISI 1045 steel.

Similar implementations were used by Arrazola et al. [95], Thakare and Nordgren [96], Liu et al. [97] and Masoudi et al. [98] to measure the temperature of tools to investigate the effect of process parameters and tool wear on the cutting temperature during the orthogonal cutting operations of various metals. In the latter study, the factors affecting the accuracy of the IR-camera measurements were also evaluated, indicating the necessity for more precise emissivity calibrations. Kryzhanivskyy et al. [82], Monica et al. [99], Jafarian et al. [100] and Liao et al. [101] used IR-camera measurements to investigate the effect of cutting temperature on the surface microstructure of the workpiece and used their findings to inform their predictive FE models.

Yang et al. [102] investigated the effect of different pure-iron grain sizes on the cutting temperature. Bjerke et al. [103] employed the IR-camera technique to monitor the tool temperature distribution during an investigation into the influence of oxygen on the degradation of tool coatings. Hao et al. [104] investigated the effect of a TiAlN-coated tool on the cutting temperature during the turning of H13 hardened steel. A reduction in cutting temperature was observed when machining with the coated tool compared to that of the uncoated tool. Menon and Madhavan [105] and Heigel et al. [37] used transparent yttrium aluminum garnet (YAG) cutting tools and a mirror alongside an IR camera to monitor the temperature at the tool–chip interface during the orthogonal cutting of a Ti-6Al-4V titanium alloy.

Solter et al. [12] used an IR camera to obtain spacial temperature measurements of the chip, tool, workpiece and their interfaces, to inform a predictive heat-partitioning FE model. Saez-de-Buruaga et al. [106] used an IR camera to measure the tool-side temperatures to calculate the tool–chip tool temperatures on the tool. The aim of this study was to determine the effect of the workpiece material, various ferrite-pearlite steels, on the tool–chip temperature to optimise the machining process for each alloy. Saleem et al. [107] used the IR camera to obtain cutting temperatures during face milling of Inconel 625. The researchers aimed to evaluate and quantify the tool life and workpiece surface integrity under various cutting parameters. A micro-thermal camera was utilised by Armendia et al. [108] to record the temperature distribution during the continuous and interrupted cutting of a Ti-6Al-4V titanium alloy and AISI 4140 steel using coated-carbide milling inserts.

**Section Findings:** Thermal cameras have evolved to be capable of high-speed thermal imaging that enables researchers to capture rapidly occurring temperature changes in the surface of the tool, workpiece and chips during machining operations. The IR-camera approach enables for non-destructive means of obtaining and analysing the temperature-distribution map of the area of interest, without affecting how heat is distributed within the materials. Furthermore, it can be consistently used without creating any mechanical wear.

Overall, IR cameras can be an effective method for temperature measurements in machining operations. However, due to limitations associated with this method, careful considerations must be made to increase the accuracy and validity of the temperature measurements. The limitations of IR cameras include the following:

- The high-speed IR cameras are an expensive investment.
- It is limited to surface temperature monitoring.
- Capable of temperature measurements of areas with a direct line of sight. In milling operations where information about the tool, or the tool–chip interface, temperature is required, an IR camera’s line sight might become obscured by swarf breaking off from the workpiece, subsequently impeding the measurement accuracy.
- The cameras must be appropriately calibrated using approximate black-body calibration sources and also by defining emissivity coefficients for a range of temperatures, as the measurements are dependent on the emissivity of the material [109,110]. In the literature reviewed, most researchers assumed the material emissivity to be a constant value, which led to the introduction of uncertainties and errors in their measurements. In reality, the emissivity of a material can change during machining due to topological changes, as well as the formation of oxide layers.
- They are unsuitable for reliable and accurate measurements in machining operations where lubricants or coolants are used due to changes in emissivity.

#### 2.2.2. Infrared Thermometers

Infrared thermometers (IRTs) operate on similar principles and execution as the IR cameras. The main difference between IRTs and IR cameras is that IRTs take single point measurements, averaging the temperature of the observed area, instead of plotting a temperature map. Lenses are utilised to focus the incoming radiation, from within the IRTs’ field of view, onto a detector where the average temperature of the area observed is measured. IRTs are often fitted with a laser as a guide to precisely locate the area of interest.

**Figure 7:**
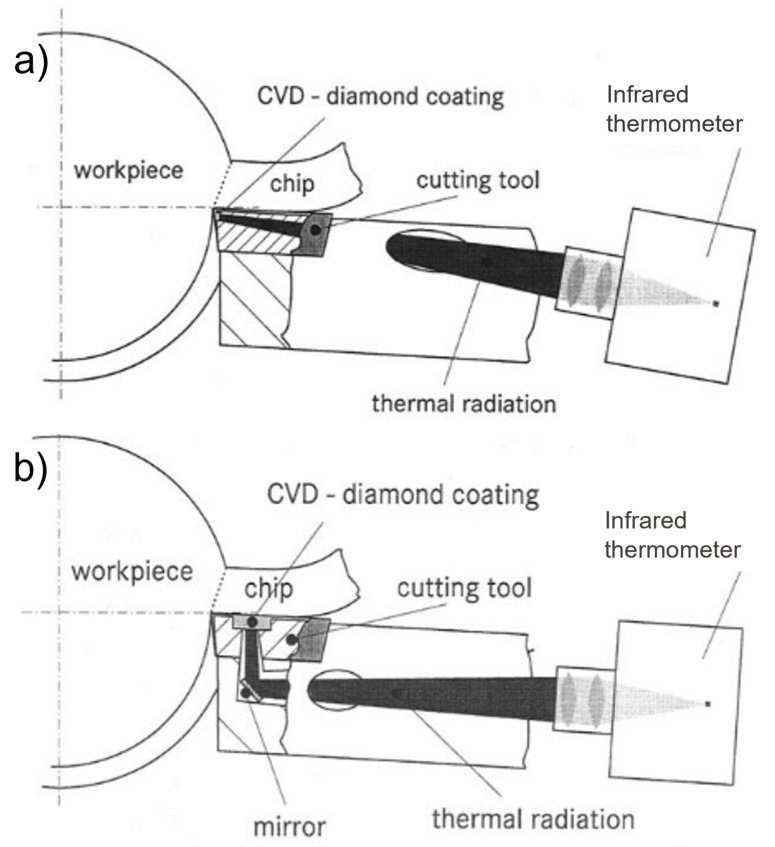
An illustration of Müller-Hummel and Lahres infrared thermometer setup (**a**) with a direct straight line of sight and (**b**) with a mirror for a direct line of sight at a right angle [111].

Muller-Hummel and Lahres [111] used an IRT to measure the temperature at two different positions of the tool–chip interface, at the tool’s flank and at the tool’s face, during turning operations. Two different modifications were made to the tool: the first required a straight borehole to be made from the edge of the tool to its side, as illustrated in Figure 7a; the second required a right angle borehole with mirrors to reflect the emitted radiation to the IRT, as illustrated in Figure 7b. A CVD diamond window was integrated onto the cutting tool to prevent the holes from getting blocked and to enable for measurements at the tool–chip interface. In addition, this technique allows for single-point temperature measurements at different depths within the material, as long as the right focusing optics are selected for an appropriately sized hole on the material. This approach may result in additional costs to the manufactured parts, as the cutting tool and tool holder require additional machining and treatments to enable tool–chip temperature measurements.

Ng et al. [44] positioned an IRT to measure the surface temperature at the back of the chip during turning operations. Similarly, Kus et al. [22], Motorcu et al. [112], Rezende et al. [113] and Kuntoğlu et al. [114] positioned their IRTs to measure the temperature at the tool–chip interface during orthogonal turning operations.

Ming et al. [115] used an IRT to monitor the workpiece surface temperature during high-speed milling. Their IRT measurements were used to inform a mathematical three-dimensional heat-conduction model. Longbottom et al. [116] used a mounting bracket to secure the IRT onto the machine quill.The IRT was pointed to measure the temperature of the new workpiece surface and the measurements were compared to those from a mathematical predictive model. Their approach was used to determine the heat partition ratio during milling operations.

**Section Findings:** IRTs are capable of accurately measuring temperature with very fast response times without requiring contact. These capabilities make IRTs suitable for high-speed machining applications, especially for interrupted machining processes, where the temperature of the tool can fluctuate at very high intervals, proportional to the cutting speed. IRTs share some of the limitations of the IR cameras, as they operate on the same principles. Their limitations include:

- A direct line of sight to the point of interest is required.
- Unable to measure the temperature gradient as they are only capable of single-point measurements.
- The detector can be sensitive to ambient temperature.
- Infrared thermometers need to be calibrated with an emissivity value to give a temperature measurement. However, the emissivity of the tool or workpiece can vary during the machining process due to the use of coolants and lubricants or the formation of oxide layers with different emissivity values.

Ratio, or two color infrared thermometers, can overcome issues with emissivity uncertainties due to obscured views. This is possible by measuring the spectral radiance of the target at two distinct wavelengths and determining the target’s temperature from the ratio of the two signals [88,117]. Zhao et al. [118] used a two-colour IRT to measure the cutting temperature profile and maximum cutting temperature of Inconel 718 with ceramic tools during turning operations. They reported results comparable with results reported in the literature and found their proposed methodology to be an appropriate and economical approach of assessing the heat generation in turning operations of Inconel 718.

The emissivity of the material at the two wavelengths measured by the detector was different. As a consequence of this, two-colour IRTs have a high sensitivity to measurement errors, which can lead to comparatively large temperature errors.

#### 2.2.3. Fibre-Optic Infrared Thermometers

A FO IRT consists of a fibre to collect and transmit the emitted radiation onto a photosensitive detector. A fiber-optic infrared thermometer can ameliorate issues with line-of-sight disruptions and emissivity uncertainties introduced by reflected radiation by placing the fibre closer to the area of interest. Furthermore, FO IRTs are capable of better protection of the electronics from the aggressive machining environment by being placed away from the target with the emitted radiation transmitted by the fibre.

Müller and Runz [119,120] used quartz fibre and two-color IRT to measure the temperature at the tool–chip interface during turning operations. A hole was made through the cutting tool, in a manner similar to that illustrated in Figure 7a, where the quartz fibre was embedded. This implementation allowed for measurements with a high sample rate with a reported response time of 1 ms and a high accuracy, which enabled the researchers to observe the transient heating-up phase, followed by the heating up due to tool wear until the cutting edge was broken in an equivalent cutting time of 2 s.

Al Huda et al. [121] utilised a two-colour IRT with an unspecified optical fibre using a translucent alumina cutting tool in an implementation similar to that of Müller and Runz [119,120]. The aim of this study was to measure the temperature distributions at the rake face of the tool under wet and dry conditions whilst turning, validating the experimental results with a FE model. It was found that the maximum temperature occurred at a distance of 0.6 mm from the cutting edge on the tool–chip interface.

A similar implementation for turning operations was employed by Tapetado et al. [122], where a silica fibre was used with a two-colour IRT. This study evaluated the effect of the distance between the fibre tip from the cutting surface on the spacial resolution. Furthermore, the influence of potential damage on the fibre tip, due to factors from the machining environment, on the output power measured by the IRT. It was demonstrated that the measured temperature was independent of the distance of the fibre as well as of any potential damage to the fiber tip.

Oezkaya et al. [123] embedded two quartz fibres symmetrically into a twist drill tool to measure the cutting temperature whilst drilling Inconel 718 under wet conditions. During the drilling process, the fibre was required to remain in contact with the cutting edge due to the high-pressure cutting-fluid supply used, resulting in the fibre being machined throughout the process. It was found that increasing the cutting force did not result in any significant temperature change; however, increasing the cutting speed from 35 m/min to 45 m/min resulted in a temperature rise of 140 °C.

Saelzer et al. [124] and Afrasiabi et al. [125] used a commercially available FO IRT to measure the temperature of the rake face during orthogonal cutting to investigate the effect of different tool surfaces on the machining temperature. The fibre was positioned perpendicularly to the tool’s rake face. To enable for rake-face temperature measurements, the researchers introduced three slots into the workpiece surface, with the distance between each slot selected to allow for a stationary temperature to be reached before the next slot. This methodology created a locally and temporarily interrupted chip with a continuous chip flow, to allow for the rake-face temperature to be measured.

Sato et al. [126,127] and Ueda et al. [58] demonstrated the potential of tool-embedded FO IRTs in milling operations. Their experimental setup consisted of two fluoride glass fibres; the first fibre ran through the inside of the tool holder and was inserted into a blind hole in the tool insert at a known depth away from the tool rake face; the second fibre was stationary, coupled to the first fibre using a non-contact fibre coupler at one end, and attached to a custom two-colour IRT and a calcium-fluoride focusing lens. The FO IRT used for their experiments was reported to have a response time of 1 ms, making it suitable for most high-speed milling operations.

The methodology employed by Sato et al. [126] and Ueda et al. [58] used a non-contact fibre coupler to help resolve the problem of manipulating thermometer wires and fibres within the rotating tool. This study demonstrated one of the best approaches for temperature measurements in milling operations, as it yielded highly accurate results with a high response time in a robust manner. The main limitations of this approach include additional complexities and costs for the customised tool spindle to accommodate the fibres and the coupler.

A different approach to eliminate issues with unknown or varying emissivity without using a two-colour IRT was proposed by Heeley et al. [55]. Their proposed approach involved embedding a sapphire fibre inside a blind hole on the cutting tool and attaching it to a small sized, custom single-colour IRT positioned onto the tool holder. By embedding the fibre inside a blind hole, the researchers created a black-body cavity where the emissivity within the cavity was approximately 0.99. Furthermore, this approach can be used in machining operations where lubricants or coolants are necessary. The authors reported a good temperature resolution at a measurement frequency of 1 kHz, corresponding to a response time of 1 ms, amongst the fastest temperature response times reported in literature. The main limitation of this approach is ensuring that the fibre is well-protected from the swarf separating from the workpiece at high velocities, as well as ensuring there are no additional stresses applied to the fibre due to vibrations. In addition, this tool-embedded approach does not measure the surface temperature; however, as with the embedded thermocouple technique, the surface temperature can be extrapolated.

Tanaka et al. [128] utilised a workpiece-embedded approach with an unspecified fibre and a two-colour IRT, to investigate the influence of cutting fluid on the tool-edge temperature for end-milling operations of a titanium alloy. The fibre was fitted through holes made in the workpiece and a pressurised air system was used to prevent foreign matter from clogging the holes, subsequently obscuring the view of the fibre. Furthermore, the fibre was moved within the hole during the milling process, so that it remained at a constant distance away from the cutting edge. Tanaka’s et al. [128] approach was an effective way of measuring the temperature of the cutting tool’s edge

Han et al. [129] embedded a multimode fibre, with a pure-silica core of 400 µm in diameter, into a cutting tool at a distance of 0.3 mm away from the cutting edge. The fibre was optically coupled to a near-infrared two-colour IRT. This study aimed to obtain real-time measurements of the temperature at the cutting edge during continuous and interrupted turning operations of AISI 316L stainless steel. Their system was able to record a maximum temperature of 679.15 °C, achieved a response time of 10 ms, and a mean square error of the measured repeated-cutting-temperature difference of 4.69 °C.

**Section Findings:** Fibre-optic infrared thermometers are capable of achieving accurate measurements with very-high response times, making them the most suitable temperature measurement technique for milling operations. FO IRTs, compared to other thermocouple techniques, can have a limited temperature range based on a combination of the properties of the selected fibre, optics and detector; however, the range can be sufficient to cover the temperature range of interest during most machining operations. The use of two-colour FO IRTs can overcome issues with emissivity uncertainties; however, they can be prone to large measurement errors. IRTs with tool-embedded fibres can ameliorate both issues with unknown emissivity, as well as issues with the large measurement errors by creating a black-body cavity with a constant emissivity. The limitation of this approach is that the surface temperature is not recorded; it is the temperature of the material at a known depth from the surface. The surface temperature can be extrapolated from the temperature recorded by the IRT.

With fibre-optic IRTs, some of the more critical challenges faced with machining can be resolved. This includes the ability to log measurements accurately with sufficiently fast response times. The fibre-optic approach can be limited by the cost and properties of the selected fibre. The operating temperature of the fibre will determine if the fibre can be embedded, as temperatures can reach 1000 °C, causing irreversible damage to the fibre. Furthermore, the rigidity and brittleness of the fibre can affect the robustness of the implementation, as the vibrations created during machining can put the fibre under stress, causing it to break. More robust FO IRT implementations are necessary to make this technique suitable for a wider range of machining operations.

## 3. Conclusions

Temperature monitoring in machining operations is of paramount importance, as it can have detrimental effects on the tool wear rate and the metallurgical properties of the workpiece, leading to an increase in waste and cost. Through the study of heat partitions, the highest temperature experienced during machining occurs at the tool–chip interface. Temperature monitoring is vital for machining operations, however, this review can conclude that there is not one single ideal temperature measurement technique for all metal machining processes. The selection of the most appropriate approach lies with individual researchers based on their experimental criteria and on what area they are interested in investigating. A comparative summary of the temperature measurement techniques for metal machining is shown in Table 1.

Measuring the temperature using workpiece-embedded methodologies can provide a great insight into temperature generation and distribution for FE modeling and research purposes but is unsuitable for industrial use. Workpiece-embedded methodologies require additional machining to make holes for the sensors, compromising the integrity of the machined component and increasing operational costs.

Turning operations are a relatively easy to implement temperature measurement methodology because the cutting tool, and subsequently the tool–chip interface where the highest temperature is expected, remain stationary. Significant complications are introduced when conceptualising the implementation of temperature monitoring in milling operations. The first complication is introduced with the discontinuous nature of milling, with a heating phase and a cooling phase, coupled with the high rotating speeds. High-speed milling applications require a temperature monitoring technique capable of acquiring temperature readings at least every 1 ms, which was only achieved through the use of radiative temperature measurement techniques, as reported in the literature. The second complication is introduced due to the rotation of the cutting tool, making the robust implementation of any temperature measurement technique challenging. For industrial applications, tool-embedded approaches, such as tool-embedded thermocouples and tool-embedded FO IRTs, have shown the highest potential. However, more research is required to make these techniques more robust and economical, to be sustainable for use in industrial settings. As the perfect technique which is suitable for all materials and processes has not yet been developed, careful considerations have to be made when selecting an appropriate technique to suit specific applications. This comparative review of thermocouple and radiative methodologies for temperature measurement in turning, drilling and milling processes can help guide future and current researchers to decide on the most appropriate method for their specific experimental scenarios.

Industry 4.0 aims to transition to a more sustainable and resilient industry, which has stimulated the need for more advanced sensor systems capable of collecting and transmitting large amounts of data in real time with the objective of increasing the degree of automation. It is this author’s opinion that the incorporation of these sensor systems in the form of wireless-smart-tool holders and high response temperature measurement methodologies, such as FO IRTs, with data-processing capabilities have the highest potential for use in the machining industry of the future. Such systems offer very fast response times which can provide sufficient measurement rates for high-speed machining applications, of which thermocouple systems are not capable. In contrast to thermocouple systems, IRTs do not drift over time and are not prone to hysteresis effects under thermal cyclic conditions, which would enable for more reliable and accurate measurements. Future developments in these approaches will see a reduction in cost, size and shape in more robust implementations to enable for better integration in high-speed drilling and milling operations to align with the goals of Industry 4.0.

## Figures and Tables

**Table 1:** Comparative summary of the temperature measurement techniques used in machining.

Techniques		Major Merits	Major Limitations
**Thermocouples**	**Tool–Work**	- Relatively simple to implement - Inexpensive	- Unsuitable for use where coolants or lubricants are required - Limited to non-indexable tools - Average temperature of the entire contact area
	**Embedded**	- Relatively simple to implement in tools for turning operations - Inexpensive - Ideal for information on heat distribution within the workpiece material	- Workpiece embedded is a destructive technique and unsuitable for manufacturing - Relatively complex implementation for milling operations - Requires tool modification for the tool-embedded approach - Surface temperature cannot be recorded - Insufficient response to rapid temperature changes
	**Single-wire**	- Suitable for experimental laboratory work - Helpful to validate FE models	- Destructive technique and unsuitable for manufacturing - Unsuitable for turning operations
**Thermal Camera**		- Very fast measurements - Temperature distribution map - Non-destructive	- Emissivity calibration uncertainties - Uninterrupted direct line of sight necessary - Unsuitable with use of lubricants or coolants
**Infrared Thermometer**		- Very fast response time - Non-destructive - High accuracy of measurements	- Emissivity calibration uncertainties - Detector sensitivity to ambient temperature
**Fibre Optic Infrared Thermometer**		- Very fast measurements - Can be used in any material and process - High measurement accuracy	- Workpiece embedded is a destructive technique and unsuitable for manufacturing - Relatively complex implementation for milling operations - Requires tool modification for the tool-embedded approach - Detector sensitive to ambient temperature
